# Autologous platelet‐rich plasma and fibrin‐augmented minced cartilage implantation in chondral lesions of the knee leads to good clinical and radiological outcomes after more than 12 months: A retrospective cohort study of 71 patients

**DOI:** 10.1002/jeo2.70051

**Published:** 2024-10-16

**Authors:** Fabian Blanke, Franziska Warth, Nicola Oehler, Johanna Siegl, Wolf Christian Prall

**Affiliations:** ^1^ Department of Knee‐, Hip‐, Shoulder‐, and Elbow Surgery, FIFA Medical Centre of Excellence Schön Klinik München Harlaching München Germany; ^2^ Department of Orthopedic Surgery University Rostock Rostock Germany; ^3^ Department of Orthopedic Sports Medicine and Arthroscopic Surgery Hessing Stiftung Augsburg Augsburg Germany

**Keywords:** autologous, knee joint, minced cartilage, PRP

## Abstract

**Purpose:**

The treatment of cartilage lesions remains a challenge. Matrix‐associated autologous chondrocyte implantation has evolved to become the gold standard procedure. However, this two‐step procedure has crucial disadvantages, and the one‐step minced cartilage procedure has gained attention. This retrospective study aimed to evaluate the clinical and radiological outcome of an all‐autologous minced cartilage technique in cartilage lesions at the knee joint.

**Methods:**

In this retrospective cohort study, 71 patients (38.6 years ± 12.0, 39,4% female) with a magnetic resonance imaging (MRI) confirmed grade III–IV cartilage defect at the medial femur condyle (*n* = 20), lateral femur condyle (*n* = 2), lateral tibia plateau (*n* = 1), retropatellar (*n* = 28) and at the trochlea (*n* = 20) were included. All patients were treated with an all‐autologous minced cartilage procedure (AutoCart™). Clinical knee function was evaluated by the Tegner score, visual analogue scale, the subjective and objective evaluation form of the International Knee Documentation Committee and the Knee Injury and Osteoarthritis Outcome Score (KOOS). MRI analyses were performed by magnetic resonance observation of cartilage repair tissue (MOCART) 2.0 knee score. Follow‐up examination was 13.7 ± 4.2 (12–24) months postoperative.

**Results:**

All clinical scores significantly improved after surgical intervention (*p* < 0.0001), especially the subgroup sports and recreation of KOOS showed clear changes from baseline in the follow‐up examination. In the postoperative MRI evaluation, 39 of 71 patients showed a complete fill of the cartilage defect without subchondral changes in 78% of the patients in the MOCART 2.0 score in the follow‐up analysis. None of the patients showed adverse effects, which are linked to the minced cartilage procedure during the time of follow‐up.

**Conclusion:**

An all‐autologous minced cartilage technique for chondral lesions at the knee joint seems to be an effective and safe treatment method with good clinical and radiological short‐term results.

**Level of Evidence:**

Level IV.

**Abbreviations**
ACL: anterior cruciate ligament
ADL: activities of daily living
ICRS: International Cartilage Regeneration & Joint Preservation Society
IKDC: International Knee Documentation Committee
KOOS: Knee Injury and Osteoarthritis Outcome Score
MACI: matrix‐associated autologous chondrocyte implantation
MOCART: magnetic resonance observation of cartilage repair tissue
MRI: magnetic resonance imaging
PACS: picture archiving and communication system
PDw‐TSE: proton‐density‐weighted–turbo spin‐echo
PRP: platelet‐rich plasma
QoL: quality of life
SD: standard deviation
VAS: visual analogue scale

## INTRODUCTION

The treatment of cartilage lesions remains a significant challenge due to the complexity of tissue, the specific requirement for successful repair and patients' activity levels and expectations. Several surgical treatment options are available depending on the size of the lesion [15, 27]. Microfracturing is a satisfying technique for small lesions with good clinical outcomes [28, 30]. However, this technique has its limitations in medium and large lesions [15, 28]. The osteochondral autograft transfer system, autologous chondrocyte implantation (ACI), matrix‐assisted chondrocyte implantation and various cartilage repair techniques using natural or synthetic scaffolds are currently the most common therapies for medium to large defects [8, 26, 27, 28, 31]. However matrix‐associated autologous chondrocyte implantation (MACI) has evolved to become the gold standard procedure for medium and large lesions due to superior tissue quality and preservation of the subchondral bone [7, 27, 28]. Nonetheless, the two‐step procedure and high perioperative costs of MACI are crucial disadvantages [17, 26]. Therefore, minced cartilage procedures have gained more attention in recent years for the treatment of cartilage lesions [5, 35, 39]. Promising results have been obtained in small and medium focal cartilage lesions at the knee joint and many surgeons have recognised the benefits of this one‐step procedure [10, 12, 22, 23, 24, 35, 40]. However, there are several different techniques of minced cartilage procedures and it is still unclear how these different techniques influence the extent of the vitality of the minced chondral cells and the achievable tissue quality radiologically and histologically, respectively [5, 12, 35]. The application of platelet‐rich plasma (PRP), autologous fibrin or biomaterials to the minced transplant is being discussed to improve cell regeneration but the data is controversial [1, 2, 3, 18, 21, 22, 40]. A device‐related minced cartilage technique with the addition of PRP and autologous fibrin represents an all‐autologous surgical option that promises good results. However, the data about this technique is small, and clinical or radiological results are lacking. Therefore, the goal of this retrospective study was to evaluate the clinical and radiological outcome of this technique in cartilage lesions at the knee joint. To the best of our knowledge, data about this topic is lacking in recent literature. We hypothesised that this all‐autologous PRP and fibrin‐augmented minced cartilage technique leads to satisfactory clinical and radiological outcome results, comparable to the already published results of established procedures such as the MACI or microfracturing technique.

## METHODS

### Study design and study group

A retrospective case series study was performed. All patients gave their informed consent and institutional review board approval was obtained (Nr. A 2024‐0046). Inclusion criteria were patient age >16 years, and an isolated focal cartilage defect grade III–IV diagnosed by magnetic resonance imaging (MRI) and arthroscopically. Subsequent surgical treatment by minced cartilage technique alone was performed. Patients with radiologically apparent degenerative joint disease, bifocal cartilage lesions >grade II to ICRS or meniscal lesions, malalignment in the knee (>3° varus or valgus), instability of the collateral/cruciate ligaments, total/subtotal resected meniscus, vascular disorders (peripheral arterial disease) or inflammatory arthritis were excluded from the study. In all patients, the minced cartilage procedure (AutoCart™, Arthrex®) was the first‐line treatment. Preoperative diagnostics included clinical examination and MRI of the affected knee. Clinical assessments were performed by two experienced orthopaedic surgeons (F.B. and N.O).

### Surgical technique and rehabilitation

#### All‐autologous PRP and fibrin‐augmented minced cartilage implantation

**Figure 1:**
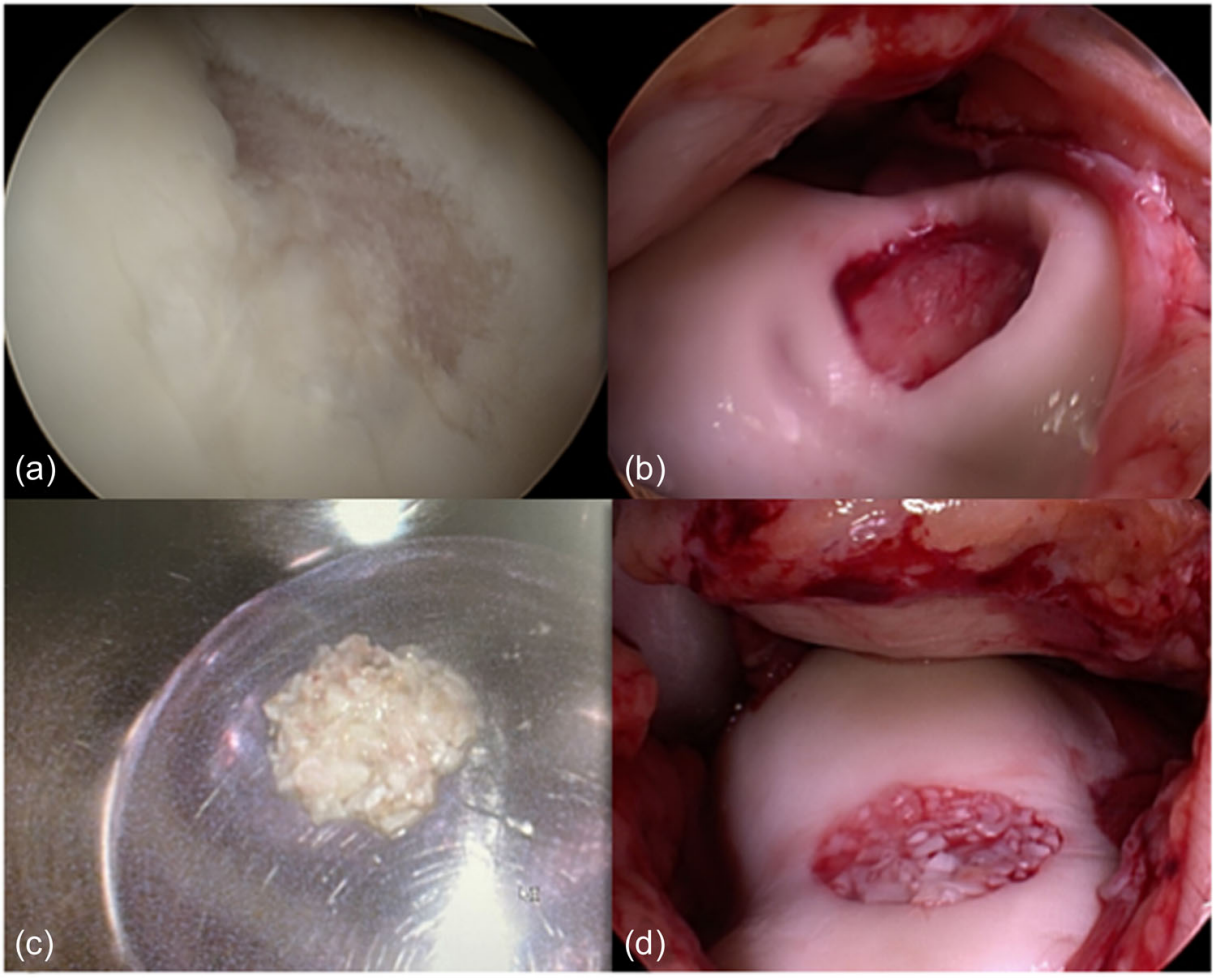
Steps of minced cartilage technique with inspection of the defect (a), debridement and tissue harvesting from the healthy rim (b), mincing and preparation of the transplant with PRP and fibrin (c) and transplantation of the whole autologous minced cartilage graft (d).

Surgical interventions were performed by two experienced surgeons (F.B. and W.C.P). The preparation of the all‐autologous PRP and fibrin‐augmented minced cartilage grafts was conducted according to the company's protocol (AutoCart™; Arthrex®). The technique was applied at the patella, trochlea and both femoral condyles. The surgical technique described in the following is for the trochlea and identical for all other locations (Figure 1). The patient's position was supine. A tourniquet was used to implant the cartilage under bloodless settings. Since autologous PRP will be required for the procedure, it is recommended to draw venous blood from the patient (e.g., cubital veins) under completely sterile conditions before initiation of anaesthesia or at the side without cannula placement (to avoid detrimental effects of narcotic substances on the PRP). It is suggested to collect at least 10–15 mL of pure PRP. The PRP (ACP, Fa. Arthrex) was then further processed during arthroscopy. Every indicated minced cartilage procedure was initiated via standard arthroscopy of the index knee joint including possible cointerventions. The intended‐to‐treat cartilage defect was well inspected, and a final indication was given during arthroscopy. The size of the defect was measured after debridement. All transplantations were performed in the mini‐arthrotomy technique. The cartilage defect was debrided in a standardised fashion by using a small sharp spoon and a ringed curette. The technique for an optimal debridement of cartilage lesions creating a stable wall and viable rim has been described in detail previously [36]. The calcified layer was gradually debrided. Microfracture or microdrilling of the subchondral bone for the influx of blood into the defect was not conducted, as contamination of the transplant by the blood clot can negatively affect the chondral fragments and does not provide a significant amount of mesenchymal stem cells [38]. The cartilage graft was harvested from the defective cartilage itself in the cases of acute traumatic chondral lesions when the cartilage clearly appeared healthy and had only lately been delaminated. Degenerative cartilage from the defect or tissue from the intercondylar notch was not utilised for further transplantation. The typical harvesting site in the present study was the healthy rim of the cartilage defect, and the minor enlargement of the defect of approximately 1 mm circumferentially was tolerated. Cartilage was harvested by use of a curette and a 3.5 shaver device. Beforehand, a collecting device (e.g., GraftNet^TM^; Arthrex®) was connected to the shaver for harvesting. In most of the cases (*n* = 69/71) the cartilage fragments were harvested after arthrotomy by a curette, collected in a water‐filled bowl and minced with the 3.5 shaver afterwards. Subsequently, the minced cartilage was mixed with 2–3 drops of ACP outside the patient in a bowl, resulting in a malleable substance. Then, 3 mL and 15–20 min later 6 mL of the ACP was inserted into a specific device (Thrombinator^TM^; Arthrex®) and was gently mixed. In the meantime, the prepared defect was inspected. Refinement of defect preparation might be performed with a curette. In the next step, thrombin that was just collected from the Thrombinator^TM^ was applied drop by drop over the chips‐paste. The minced cartilage/ACP/thrombin‐paste was distributed over the defect for complete coverage in an open technique. The consistency of the paste provided initial stability, as the thrombin combined with the ACP activates fibrin, which stabilises the minced cartilage graft and fixes it to the defect site. Additional fibrin was generated by mixing the remaining thrombin and ACP in a ratio of 1:1. The additional fibrin was used to finally seal the implanted minced cartilage graft. The filling of the treated defects with the cartilage transplant achieved at least 80% of the height of the surrounding healthy cartilage in all cases. The procedure was then finished. A drain was not applied. The leg was placed into full extension and immobilised in a straight brace. The straight brace was changed to a hinged knee brace after 2–7 days. Partial weight‐bearing of 10 Kg was performed for 2–6 weeks due to the location of the lesion.

### Clinical and radiological outcome scores/MRI evaluation

All patients were evaluated preoperatively and at 12–24 months follow‐up, including physical examination and completion of a questionnaire. Postoperative complications or adverse effects that needed medical attention in time between surgery and final follow‐up were recorded retrospectively. The subjective and functional scores applied to evaluate the clinical outcome included the Tegner Score, visual analogue scale (VAS) for pain at daily activities, and the subjective and objective evaluation form of the International Knee Documentation Committee (IKDC). Also, the Knee Injury and Osteoarthritis Outcome Score (KOOS) assessing knee pain, symptoms, activities of daily living (ADL), sports and recreation, and knee‐related quality of life (QoL) was assessed. The KOOS score, IKDC score and Tegner Activity Scale are verified tools for assessing knee function and activity levels in patients with knee injuries, including cartilage lesions, and were validated in the native language of the patients [14]. The KOOS covers five subscales (Pain, Symptoms, ADL, Sport/Rec, and QOL) and scores range from 0 to 100, with higher scores indicating better outcomes [32]. The IKDC score, also ranging from 0 to 100, evaluates symptoms, function and sports activity, where higher scores denote better knee function [20]. The Tegner Activity Scale ranges from 0 to 10, reflecting activity levels from sick leave to elite competitive sports, with higher scores indicating higher activity levels [14]. All three scales have been translated and validated in English, with minimal clinically important differences established to help interpret changes in patient conditions, such as improvements of 7–12 points for KOOS subscales, 11–20.5 points for IKDC and a 1‐level improvement for the Tegner scale being clinically significant. MRI of the respective knee joint was conducted before surgery and at 12–24 months final follow‐up. Since MRI scans were part of the routine clinical follow‐up and not conducted for a prospective study, sequence parameters varied slightly. Yet, the majority of the patients (*n* = 59) underwent an MRI examination on the same 3.0‐Tesla scanner (Avanto; Siemens Medical Systems) using an 8‐channel phased‐array extremity coil. The following sequences were performed: [1] sagittal fat‐saturated (fs) proton‐density‐weighted turbo spin‐echo (PDw TSE) sequence [2], a sagittal T1‐weighted TSE [3], a coronal fs PDw TSE sequence and for patients with trochlear chondral defects and [4] an axial fs PDw TSE sequence, respectively. The MRI scans were evaluated using the Infinitt PACS viewer (Infinitt Europe GmbH). The size of the lesions was determined with the PACS measuring tool if the size of the defect was not described in the surgery report. MRI scans were analysed using the MOCART (Magnetic Resonance Observation of Cartilage Repair Tissue) 2.0 Knee Score, a recently published update on the original MOCART score [4]. This revised version integrates seven variables (volume fill of cartilage defect, integration into adjacent cartilage, surface of the repair tissue, structure of the repair tissue, signal intensity of the repair tissue, bony defect or bony overgrowth, subchondral changes). Overall, a score ranging from 0 to a maximum of 100 points may be reached. MRI evaluations were performed by two independent experienced clinicians with musculoskeletal subspeciality. After an initial blinded assessment, all images were reviewed in consensus.

### Statistical analysis

Statistical analysis was performed using GraphPad Prism 7 software (GraphPad software). Continuous and categorical variables were expressed as mean ± standard deviation (range) and n, respectively. Comparisons of clinical scores from baseline to follow‐up were analysed using paired Student's t‐test. Comparisons of clinical and MOCART score changes from baseline concerning gender were performed using an unpaired *t*‐test. ANOVA‐test was used for the comparison of clinical and radiological score changes from baseline between distinct defect localisations. For correlation analysis between defect size and age, and radiological and clinical scores, respectively, Spearman coefficient ρ was used. *p* < 0.05 were considered statistically significant.

## RESULTS

From 102 patients with a grade III–IV cartilage defect and treatment by the whole autologous minced cartilage technique, 71 patients with a mean age of 38.6 y ± 12.0 (15–62) were included between April 2021 and December 2023. Forty‐two lesions (59%) were classified as traumatic due to MRI findings and medical anamnesis. Thirty‐one patients were excluded due to leg malalignment (*n* = 17), ACL instability (*n* = 7), bifocal lesions (*n* = 4) or meniscal lesions (*n* = 3). In all 71 patients, minced cartilage procedures were performed alone. Detailed patient characteristics are displayed in Table 1.

**Table 1:** Demographic data and baseline characteristics.

Patients (*n*)	71
Gender (male/female, *n*)	43/28
Age (years, mean ± SD, range)	38.6 ± 12.0 (15–62)
BMI (mean ± SD, range)	25.75 ± 3.03 (20.7–30.5)
Follow‐up (months, mean ± SD, range)	13.7 ± 4.2 (12–24)
Side affected (left/right, *n*)	36/35
Defect size (cm^2^, mean ± SD, range)	4.5 ± 1.9 (2.0–10.0)
Defect size distribution (*n*)	
<3 cm^2^	11
3–5 cm^2^	44
>5 cm^2^	16
Defect localisation (*n*)	
Medial femur condyle	20
Lateral femur condyle	2
Medial tibial plateau	0
Lateral tibial plateau	1
Retropatellar	28
Trochlea	20

**Figure 2:**
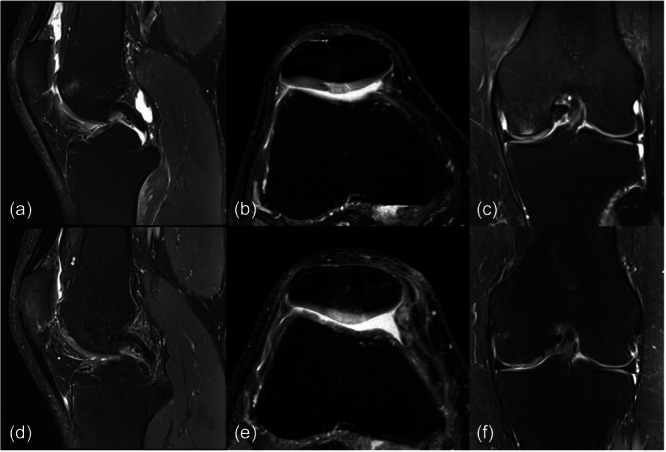
Preoperative MRI (a–c) of chondral defects in the trochlea (a), retropatellar (b) and at medial condyle (c). Corresponding postoperative MRI after the minced cartilage procedure (d: 14 months, e: 12 months, f: 24 months).

All clinical scores significantly improved after surgical intervention. IKDC score increased from 42.3 ± 20.5 to 83.1 ± 12.6 and VAS pain at daily activities decreased from 4.9 ± 1.0 to 1.3 ± 1.7 at the final follow‐up (*p* < 0.0001). Moreover, QoL showed an improvement from 34.7 ± 21.8 to 63.4 ± 25.4 and Sports and recreation from 22.1 ± 31.5 to 81.1 ± 18.8, respectively, in the KOOS at the final follow‐up (*p* < 0.0001). (Table 2). In the postoperative MRI evaluation, 39 of 71 patients showed a complete fill of the cartilage defect without subchondral changes in 78% of the patients in the MOCART 2.0 score in follow‐up analysis (Table 3, Figure 2). When correlation analysis was performed, clinical scores and MOCART 2.0 score did not display positive or negative associations with age or defect size. Also, the comparison between female and male patients did not display statistically significant differences in clinical or radiological outcome scores apart from one subgroup. Of all distinct scores, only the ADL subgroup of KOOS score showed higher change from baseline values for male patients compared to female patients (*p* = 0.004). Comparison of clinical and MOCART 2.0 score between femorotibial, patellar or trochlear defect localisation did not display any statistically significant differences apart from the ADL subgroup of KOOS score (*p* = 0.03) (Table 4). Ten of 71 patients showed minor complications, such as delayed wound healing (*n* = 5) moderate hemarthrosis at discharge with restricted flexion after three months postoperative (*n* = 3), deep vein thrombosis (*n* = 1) and sensibility disorder at the lateral knee compartment (*n* = 1), which did not require surgical interventions. None of the patients showed adverse effects which are linked to the minced cartilage procedure during the time of follow‐up.

**Table 2:** Evaluation of clinical outcome according to the distinct scores (VAS, Tegner, IKDC, KOOS) before surgery and at follow‐up examination.

Score	Minced Cartilage						*p* value
	Baseline		Follow‐up		Change from baseline		
	Mean ± SD	Range	Mean ± SD	Range	Mean ± SD	Range	
VAS	4.9 ± 1.0	3.0 to 7.0	1.3 ± 1.7	0.0 to 5.0	3.6 ± 2.0	0.0 to 7.0	<0.0001
Tegner	2.3 ± 0.7	1.0 to 4.0	4.8 ± 1.2	3.0 to 7.0	2.3 ± 1.8	−4.0 to 6.0	<0.0001
IKDC	42.3 ± 20.5	19.5 to 75.9	83.1 ± 12.6	51.7 to 100.0	39.6 ± 27.9	−67.0 to 77.0	<0.0001
KOOS							
Pain	60.9 ± 19.1	25.6 to 94.5	89.2 ± 12.0	33.0 to 100.0	27.5 ± 23.8	−72.2 to 68.9	<0.0001
Symptoms	64.2 ± 17.9	7.4 to 93.9	85.8 ± 10.8	64.3 to 100.0	20.4 ± 23.5	−93.5 to 78.9	<0.0001
ADL	65.0 ± 20.8	18.5 to 100.0	95.1 ± 9.3	46.3 to 100.0	28.7 ± 25.8	−71.8 to 81.4	<0.0001
Sports and recreation	22.1 ± 31.5	0.0 to 100.0	81.1 ± 18.8	10.0 to 100.0	57.9 ± 38.65	−65.0 to 100.0	<0.0001
QoL	34.7 ± 21.8	5.75 to 81.0	63.4 ± 25.4	18.5 to 100.0	27.9 ± 36.8	−56.0 to 96.0	<0.0001

Abbreviations: ADL, activities of daily living; IKDC, International Knee Documentation Committee; KOOS, Knee Injury and Osteoarthritis Outcome Score; QoL, quality of life; VAS, Visual analogue scale for pain at daily activities.

**Table 3:** MOCART 2.0 score: Overall and distribution according to subdomains.

MOCART 2.0 score subdomains (points)
Volume fill of cartilage defect (0–20)
Integration into adjacent cartilage (0–15)
Surface of the repair tissue (0–10)
Structure of the repair tissue (0–10)
Signal intensity of the repair tissue (0–15)
Bony defect or bony overgrowth (0–10)
Subchondral changes (0–20)
**Total (0–100)**

Abbreviations: MOCART, Magnetic Resonance Observation of Cartilage Repair Tissue; SD, standard deviation.

**Table 4:** Comparison of clinical and radiological outcome scores (VAS, Tegner, IKDC, KOOS) according to location of cartilage lesion.

Change from baseline	Femorotibial		Patellar		Trochlear		*p* value
	Mean ± SD	Range	mean ± SD	Range	Mean ± SD	Range	
VAS	3.8 ± 2.0	0.0 to 6.0	4.0 ± 1.7	0.0 to 7.0	2.9 ± 2.3	0.0 to 6.0	ns
Tegner	2.4 ± 1.3	0.0 to 5.0	1.8 ± 2.3	−4.0 to 5.0	2.9 ± 1.6	0.0 to 6.0	ns
IKDC	44.0 ± 22.0	0.0 to 77.0	40.3 ± 32.7	−69.0 to 77.0	33.6 ± 26.7	−10.4 to 74.7	ns
KOOS							
Pain	31.4 ± 19.9	−2.2 to 66.7	24.3 ± 28.2	−72.2 to 68.9	25.9 ± 21.6	−18.9 to 57.8	ns
Symptoms	22.7 ± 19.4	−10.7 to 72.5	19.1 ± 30.8	−93.5 to 78.9	19.7 ± 15.5	−4.0 to 40.1	ns
ADL	27.5 ± 15.4	−3.6 to 61.5	29.4 ± 32.0	−71.8 to 81.4	29.1 ± 27.0	−5.3 to 81.3	0.0302
Sports and recreation	61.5 ± 35.1	−15.0 to 100.0	55.2 ± 43.2	−65.0 to 100.0	57.5 ± 37.3	−5.0 to 100.0	ns
QoL	29.7 ± 33.9	−49.3 to 87.0	27.9 ± 38.6	−56.0 to 93.5	25.7 ± 39.2	−44.3 to 94.0	ns
MOCART	75.2 ± 13.9	60.0 to 100.0	75.7 ± 11.4	60.0 to 100.0	78.8 ± 9.4	65.0 to 100.0	ns

Abbreviations: ADL, activities of daily living; IKDC, International Knee Documentation Committee; KOOS, knee injury and osteoarthritis outcome score; MOCART, magnetic resonance observation of cartilage repair tissue; QoL, quality of life; VAS, visual analogue scale.

## DISCUSSION

The most important finding of this study was that an all‐autologous PRP and fibrin‐augmented minced cartilage implantation seems to be an effective and safe treatment method in symptomatic chondral lesions at the knee joint with good clinical and radiological results after more than 12 months postoperative.

The minced cartilage procedure is a promising one‐step technique, which showed satisfactory clinical and radiological outcome results in small chondral lesions independently from the defect location [11, 12, 13, 24, 37]. This technique can be performed arthroscopically and by arthrotomy without external tissue engineering [35, 38]. Therefore, it is an “in situ tissue engineering technique” with low costs and can be performed spontaneously if a chondral defect is detected. The technique was first described in the early 1980s and modified by different authors [35]. Recent studies showed good clinical results with improvement in clinical scores and satisfying radiological outcomes [9, 13, 24, 33, 35, 37]. However, there are some crucial doubts, which are linked to the minced cartilage technique. First of all the harvesting of the cells is not always easy and might be contaminated with necrotic or damaged chondral cells [2, 42]. Moreover, it is not clear which quality of tissue can be achieved in the long‐term follow‐up. There is evidence that viable chondral cells can be preserved by cutting the cartilage quickly and sharply in the smallest possible chips with a scalpel or special shaving blade [6, 37]. The tissue quality confirmed by histological examination was comparable to ACI and superior to microfracturing in animal models [12, 16, 25, 37]. Nonetheless, treatment of symptomatic chondral lesions was critically assessed because the data about this procedure is limited and the matrix‐associated chondrocyte implantation represents a proven technique for all sizes of chondral lesions in an open or arthroscopic approach and with verified information about the vital cell proportion in the transplant [27, 28, 37]. However, the high costs and documentation expenditure of external tissue engineering and the requests of patients for a one‐step procedure led to growing reservations about the MACI procedure [17, 24]. Thus, the minced cartilage techniques are attractive surgical options, but several different techniques of minced cartilage procedures are described, and it is still unclear how these different techniques influence the extent of vitality of the minced chondral cells and the achievable tissue quality radiologically and histological, respectively. Some techniques just use the minced cartilage chips for transplantation, other techniques add biomaterials [1, 27, 30, 35]. The minced cartilage technique of the present study adds PRP and autologous fibrin and converts the minced cartilage technique to an all‐autologous technique with the use of modern autologous biological agents. The present study showed promising clinical results after more than 12 months and good radiological outcomes with complete fill of the cartilage defect in 55% of patients and without subchondral changes in 56 of the 71 patients in the MOCART2.0 scores. All clinical scores significantly improved after surgical intervention, although it must be noted with some reservations that not all the scores used (KOOS, Tegner) have been validated for cartilage lesions, thus limiting their conclusiveness. However, the results still suggest that this minced cartilage technique works effectively in focal lesions at the knee joint and the clinical and radiological results were similar or even better compared to other minced cartilage techniques or the MACI technique [7, 13, 24, 37]. This fact might be due to the use of PRP and autologous fibrin because there is evidence that these agents can influence the migration of chondrocytes and cartilage regeneration [19, 29, 34, 41]. Moreover, in present study the cartilage tissue was harvested exclusively from the healthy edge of the defect, which may have had an positive impact on the achieved tissue quality and the implantation of necrotic cartilage tissue was prevented. However, more results that might support these assumptions about the location of tissue harvest and the advantages of this all‐autologous minced cartilage technique are still missing in the literature, and therefore, definite conclusions cannot be drawn. Thus, the present study results only represent initial evidence that this technique is safe and with promising clinical and radiological results after more than one year. However, long‐term results are needed to validate these observations.

The present study certainly has several limitations. First, there was no matched control group, which makes it difficult to securely evaluate the superior clinical effectiveness of the technique. Second, MRIs were performed at different follow‐ups and with different protocols. Moreover, the follow‐up restricts the ability to draw any conclusions on the superiority of other chondrocyte transplant procedures in the long‐term outcome. Third, all procedures were performed in a mini‐open approach, which is not described as the standard approach by the company (Fa. Arthrex) and there were no histological biopsies performed to evaluate the achieved tissue quality of the described technique. Lastly, this study did not clarify whether large cartilage lesions (>6 cm^2^) can be treated sufficiently by the minced cartilage technique or need to be enhanced by biomaterials or autologous agents. However, data about the minced cartilage technique is limited, especially about the whole autologous procedure in combination with PRP. Therefore, the data of the present study can add important knowledge to the topic of surgical cartilage treatment.

## CONCLUSION

All‐autologous PRP and fibrin‐augmented minced cartilage implantation seems to be an effective and safe treatment method for symptomatic chondral lesions at the knee joint with good clinical and radiological short‐term results.

## AUTHOR CONTRIBUTIONS

**Fabian Blanke**: Conceptualisation; data curation; formal analysis; investigation; visualisation; writing and editing. **Franziska Warth**: Conceptualisation; investigation; data curation; project administration. **Nicola Oehler**: Data curation; statistical analysis; writing, review and editing. **Johanna Siegl**: Data curation; investigation; project administration; visualisation. **Wolf C. Prall**: Wwriting, review and editing.

## CONFLICT OF INTEREST STATEMENT

The authors declare no conflict of interest.

## ETHICS STATEMENT

The study was conducted in accordance with the Declaration of Helsinki and approved by the Institutional Review Board of the University of Rostock (No. A 2024‐0046).

## Data Availability

The data underlying this article will be shared on reasonable request to the corresponding author.
